# Applying Natural Language Processing to Understand Motivational Profiles for Maintaining Physical Activity After a Mobile App and Accelerometer-Based Intervention: The mPED Randomized Controlled Trial

**DOI:** 10.2196/10042

**Published:** 2018-06-20

**Authors:** Yoshimi Fukuoka, Teri G Lindgren, Yonatan Dov Mintz, Julie Hooper, Anil Aswani

**Affiliations:** ^1^ Department of Physiological Nursing/Institute for Health & Aging School of Nursing University of California, San Francisco San Francisco, CA United States; ^2^ School of Nursing Rutgers University Newark, NJ United States; ^3^ Department of Industrial Engineering and Operations Research University of California, Berkeley Berkeley, CA United States; ^4^ Institute for Health & Aging School of Nursing University of California, San Francisco San Francisco, CA United States

**Keywords:** mobile apps, physical activity, fitness trackers, women, maintenance, accelerometer, randomized controlled trial, motivation, barriers, behavioral change

## Abstract

**Background:**

Regular physical activity is associated with reduced risk of chronic illnesses. Despite various types of successful physical activity interventions, maintenance of activity over the long term is extremely challenging.

**Objective:**

The aims of this original paper are to 1) describe physical activity engagement post intervention, 2) identify motivational profiles using natural language processing (NLP) and clustering techniques in a sample of women who completed the physical activity intervention, and 3) compare sociodemographic and clinical data among these identified cluster groups.

**Methods:**

In this cross-sectional analysis of 203 women completing a 12-month study exit (telephone) interview in the mobile phone-based physical activity education study were examined. The mobile phone-based physical activity education study was a randomized, controlled trial to test the efficacy of the app and accelerometer intervention and its sustainability over a 9-month period. All subjects returned the accelerometer and stopped accessing the app at the last 9-month research office visit. Physical engagement and motivational profiles were assessed by both closed and open-ended questions, such as “Since your 9-month study visit, has your physical activity been more, less, or about the same (compared to the first 9 months of the study)?” and, “What motivates you the most to be physically active?” NLP and cluster analysis were used to classify motivational profiles. Descriptive statistics were used to compare participants’ baseline characteristics among identified groups.

**Results:**

Approximately half of the 2 intervention groups (Regular and Plus) reported that they were still wearing an accelerometer and engaging in brisk walking as they were directed during the intervention phases. These numbers in the 2 intervention groups were much higher than the control group (overall *P*=.01 and *P*=.003, respectively). Three clusters were identified through NLP and named as the Weight Loss group (n=19), the Illness Prevention group (n=138), and the Health Promotion group (n=46). The Weight Loss group was significantly younger than the Illness Prevention and Health Promotion groups (overall *P*<.001). The Illness Prevention group had a larger number of Caucasians as compared to the Weight Loss group (*P*=.001), which was composed mostly of those who identified as African American, Hispanic, or mixed race. Additionally, the Health Promotion group tended to have lower BMI scores compared to the Illness Prevention group (overall *P*=.02). However, no difference was noted in the baseline moderate-to-vigorous intensity activity level among the 3 groups (overall *P*>.05).

**Conclusions:**

The findings could be relevant to tailoring a physical activity maintenance intervention. Furthermore, the findings from NLP and cluster analysis are useful methods to analyze short free text to differentiate motivational profiles. As more sophisticated NL tools are developed in the future, the potential of NLP application in behavioral research will broaden.

**Trial Registration:**

ClinicalTrials.gov NCT01280812; https://clinicaltrials.gov/ct2/show/NCT01280812 (Archived by WebCite at http://www.webcitation.org/70IkGagAJ)

## Introduction

Regular physical activity is associated with reduced risk of chronic illnesses, such as hypertension, type 2 diabetes, and several types of cancers [1-6]. Despite various types of successful physical activity interventions, maintenance of activity over the long term is extremely challenging [7]. In fact, approximately half of individuals who start a physical activity program will relapse or return to their previous inactive lifestyle within the first 6 months [8]. Given the high prevalence of relapse, understanding factors associated with increasing and maintaining physical activity is critical for women and racial or ethnic minority groups who have a higher prevalence of physical inactivity [9,10]. In a recent systematic review and meta-analysis, motivation and goals followed by belief about consequences and self-report of good or excellent health status are the strongest predictors of physical activity maintenance [8]. A motivational profile determines the reason(s) for one's actions, desires, and needs, and can be multi-dimensional and complex. Furthermore, this profile can be dynamic and fluctuate over time [11] based on one’s experiences, like going through a physical activity program. However, data related to long-term maintenance after cessation of an intervention is still limited.

As smartphone ownership has significantly increased in the past 10 years, (77% in 2018 in the US) [12], the use of digital technologies (ie, smartphone apps and activity trackers) to promote physical activity has gained popularity. These technologies allow investigators to incorporate critical components of physical activity maintenance like self-motivation, goal setting, and self-efficacy, to one’s daily life [7,13]. A recent systematic review has shown that smartphone apps and accelerometer-based interventions appear to improve physical activity and sedentary behaviors for at least a short period of time [14]. However, few clinical trials involving digital technologies to increase physical activity have examined sustainability of these interventions over time.

To fill this knowledge gap, we recently completed the mobile phone—based physical activity education (mPED) study, a randomized controlled trial (RCT) designed to examine the efficacy of a 3-month mobile app and accelerometer-based physical activity intervention and a 6-month maintenance intervention for physically inactive women. In this paper, semi-structured interview data collected at a 12-month telephone interview (study exit) were analyzed by natural language processing (NLP), a field of computer science which incorporates artificial intelligence and computational linguistics [15] to formulate algorithms used to extract information from textual inputs. Use of NLP in clinical and medical research began to appear in the 1980s, primarily by applying it to electronic health records (EHRs) [16-19], while NLP was brought into broader use more recently [20]. However, its application to behavioral research is still in its infancy. Therefore, to the best of our knowledge, this is the first study to use NLP to explore interview data to identify key motivational elements.

The aims of this paper are to 1) describe physical activity engagement post-intervention, 2) identify motivational profiles using NLP, and clustering techniques in a sample of women who completed the physical activity intervention, and 3) compare sociodemographic and clinical data among these identified cluster groups [15,17,18].

## Methods

### Study Design and Participants

The mPED trial is a randomized controlled trial (RCT) with 3 groups. In this paper, we analyzed the 12-month telephone interview (study exit) data of the mPED trial. Supplement 1 describes an overall study design. The primary outcome in this mPED trial was accelerometer recorded physical activity (average daily steps) over the 9-month period. Overall, the 3-month intervention resulted in a significant increase in physical activity (Regular and Plus groups versus Control group), but physical activity during the 6-month maintenance period did not significantly differ between the Regular and Plus groups.

The study protocol was approved by the University of California, San Francisco Committee on Human Research (CHR) and the mPED Data and Safety Monitoring Board. Detailed description of the study design and inclusion or exclusion of the study participants has previously been published [21-23]. In short, community dwelling physically inactive women age 25 to 65 with a body mass index (BMI) of 18.5–43.0 kg/m^2^ who do not have medical conditions or physical problems that require special attention in an exercise program were recruited from the San Francisco Bay Area between May 2011 and April 2014.

### Summary of a 3-Month Physical Activity and 6-month Maintenance Intervention

A total of 210 women were randomized into 1 of the 3 groups after completion of the run-in period. The control group received an accelerometer for 9 months but did not receive any physical activity intervention. The Regular and Plus groups received an accelerometer, an identical physical activity trial app developed by the investigator, and brief in-person sessions for the first 3 months after randomization. While the study trial app was removed from the Regular group at the 3-month visit, the Plus group kept the trial app and was encouraged to continue using the physical activity diary in the app for the remaining 6-month maintenance period. Both groups also kept an accelerometer for 9 months. At the 9-month visit, participants in all groups returned the accelerometer (and study mobile phone with app for the Plus group) to the research staff. If the study app was installed on a participants’ phone, it was removed by the research staff. Participants were encouraged to obtain and wear a pedometer/activity tracker/accelerometer to maintain their physical activity after the 9-month visit. Since the accelerometer used in the study was not commercially available, a research staff provided a list of pedometer/activity tracker/accelerometers and prices to participants who did not own one of these devices.

### Procedures of 12-month Telephone Interview and Data Collection

Research staff scheduled a 12-month follow-up telephone intervention at the end of the 9-month visit. Participants then received a text, email or telephone call to confirm their 12-month appointment, and a list of interview questions was mailed or emailed to participants prior to their interviews. After completion of the 12-month telephone interview, participants received a check in the amount of US $40. The 12-month interview consisted of two parts: 1) a survey and 2) a semi-structured, telephone interview consisting of open-ended questions. This paper focuses on the survey data.

### 12-month Telephone Interview Survey

The survey consists of 2 types of questions: 1) close-ended questions and 2) open-ended questions to assess the use of digital technologies and maintenance of physical activity, such as “What type of phone do you have?”; “Do you currently have a health-related mobile app?”; “Do you have your own pedometer?”; “Do you currently wear a pedometer?” Self-reported physical activity level and types of physical activity were assessed by the question: “Since your 9-month visit, what types of exercise have you engaged in to be physically active?” A list of exercise types was provided to participants. Additionally, participants were asked the following question, “Since your 9-month study visit, has your physical activity been more, less, or about the same as compared to the first 9 months of the study?” To assess participants’ motivation to maintain physical activity after the intervention, the research staff asked the following open-ended question: *“* What motivates you the most to be physically active?*”* They were encouraged to list at least two motivational reasons. Responses were transcribed by research members during or immediately after the interview. Later, all transcriptions were reviewed, and all typos and errors were corrected before analysis.

### Analytic plans

#### Natural Language Processing, K-Means Clustering, and Principal Component Analysis

Motivational profiles for each of the participants were generated using machine learning. First, participants’ responses to the open-ended question “What motivates you the most to be physically active?” were converted into numerical vectors that quantify responses. The numerical vectors were constructed by averaging 1000-dimensional word-vectors generated by a word2vec model trained on the Wikipedia corpus using a bag-of-words method by first converting each word in a participants’ response into an equivalent word-vector and then averaging the resulting vectors. Word-vectors were generated using a skip-gram word2vec model [24,25] trained on the data of a Wikipedia data dump from 2015 [26], common words like “and” and “the” were removed by using the stop-word set in the Natural Language Toolkit (NLTK) software package [27], and the word-vector model itself was implemented using the Genism Python package [28]. Unlike traditional statistical approaches, selection of corpus (a large collection of texts) is extremely critical in an NLP analysis. To our knowledge, a Wikipedia data dump is one of the largest open source available corpora. Second, K-means clustering [29] was performed on the numerical vectors (which are a quantitative representation of participants’ responses) using sci-kit learn [30]. The number of clusters used in the K-means clustering was derived using the elbow criterion [29]. Then, Principal Component Analysis (PCA) was used to reduce the dimensionality of the data to visualize the resulting clusters in two dimensions [29]. PCA preserves linear relationships and large distances between data points. For example, if two data points are widely separated, then they will also be widely separated in the PCA projection. The analysis was conducted on a Windows 7 laptop with a 2.4Ghz processor and 16GB of RAM, using Python 3.5.2 and Anaconda.

#### Other Analyses

Chi-square test or Analysis of Variance (ANOVA) was used to compare the sample baseline characteristics among identified cluster groups and responses to survey questions among the Control, Regular, and Plus groups. To ensure that the sample of 203 participants was sufficiently large to conduct these analyses, we performed post hoc power analysis for the ANOVA and chi-squared comparisons across the 3 motivational groups. This analysis showed that the minimum observed power obtained by our comparisons is 0.71 for this sample size and group distribution, which would indicate that the sample size is sufficient to generalize these conclusions for the study population. All survey data were entered into the software program using a double-data entry system. *P* values less than a Bonferroni-corrected .017 were considered statistically significant.

## Results

### Baseline Sociodemographics

Of those randomized 210 participants, 203 (97%) completed a 12-month survey. Mean participant age was 52.6 (SD 11.0) years, 56.7% self-identified as non-Hispanic White, and 74.4% had a full or part time job. Age, race or ethnicity, education, annual household income, marital status, and employment status did not differ between 3 treatment groups (Control, Regular, and Plus; overall *P*>.05).

### Use of Digital Technologies and Self-Reported Sustainability of Physical Activity at 12 months

At 12 months, 41.4% (84/203) of participants reported that they currently had at least 1 health-related app on their mobile phones, but this prevalence did not differ among the 3 treatment groups (*P*>.05; Table 1). While 61.6% (125/203) of the study participants reported that they owned a pedometer, physical activity tracker, or accelerometer, only 41.4% (84/203) reported they currently wore it. Use of pedometer or physical activity tracker/accelerometer in the Regular and Plus groups was significantly higher than in the Control group (52.2% and 46.2% versus 26.1%; overall *P*=.005). Among 38.1% (78/203) participants who did not have a pedometer or physical activity tracker/ accelerometer at the 12-month interview, 13.8% (28/203) reported that they were still looking for or planning to purchase one soon, and 8.4% (17/203) reported that they were too expensive to purchase or that they were going through financial difficulties.

In response to the question “Has your physical activity been more, less, or about the same compared to the first 9 months of the study?” a significantly higher proportion of participants in the Control group, compared to the Regular and Plus groups, reported engaging in more physical activity from 9 to 12 months (overall *P*=.006). However, a greater proportion of participants in the Regular and Plus groups engaged in more brisk walking compared to the Control group (overall *P*=.003). Among the 36% (73/203) of women who reported “less active,” “lack of time” (work or school cited as the main time constraint), “study ended,” and “did not have a pedometer” were the most frequently reported reasons. The proportion of women who reported lack of time and study ended in the Regular and Plus groups were statistically greater than the Control group (*P*=.02 and *P*=.04 respectively).

### Profiles of Motivation to Be Active Using Natural Language Processing and K-Mean Clustering Techniques.

**Figure 1:**
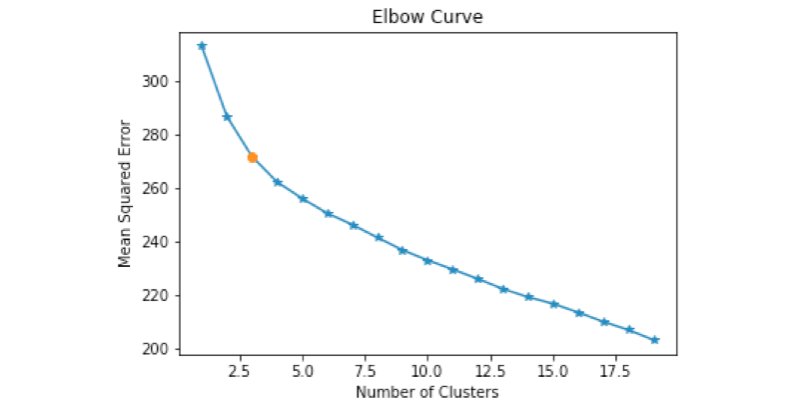
Elbow curve used to determine the number of clusters to be used in K-means clustering. On the x-axis are the number of clusters which the algorithm was set to fit and on the y-axis is the mean squared error of the clustering. The red dot is located at the mark which corresponds to 3 clusters and corresponds to the closest number of clusters to the “bend” of the elbow curve.

**Figure 2:**
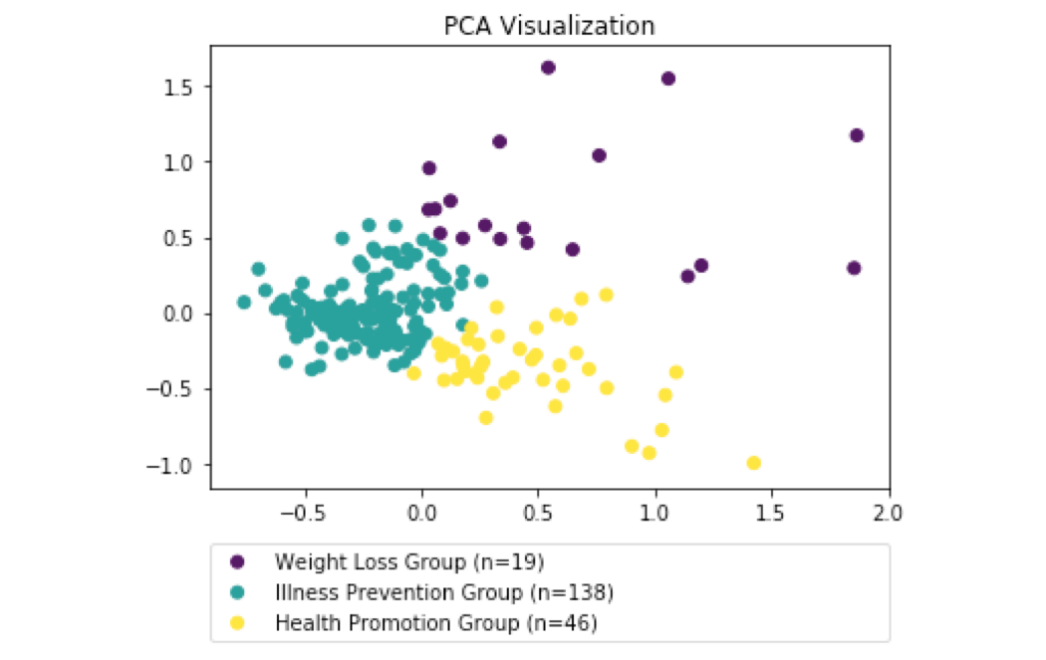
Principal Components Analysis (PCA) Visualization of motivational profiles. The plot axes represent the first two principal components of the bag-of-words vector representations of the motivations given by patients. The purple cluster corresponds to the responses of patients who listed weight loss as their sole motivation for physical activity, the teal cluster corresponds to patients who were primarily motivated by illness prevention, and the yellow cluster corresponds to those patients primarily motivated to do physical activity due to health promotion.

Overall, the top 3 most commonly used words (which are not stop words, like "the" or "and") are: "health" (n=67), "weight" (n=66), and "better" (n=65). Numerical vectors that quantify participants’ response to the question “What motivates you the most to be physically active?” were constructed by averaging 1000-dimensional word-vectors generated by the Wikipedia trained word2vec model (excluding common words like “and” and “the”). The elbow criterion was used to determine the number of clusters to set in the K-means clustering, and the resulting elbow curve is shown in Figure 1. Using this method, we determined that 3 clusters are most suitable to partition the motivational profiles effectively. Figure 2 shows the result of the Principal Components Analysis (PCA), “a statistical procedure that uses an orthogonal transformation to convert a set of observations of possibly correlated variables into a set of values of linearly uncorrelated variables called principal components” [33].

As seen in Figure 2, the 3 clusters are very distinct groups. Using these 3 clusters, we performed post-hoc qualitative analysis to define cluster names based on the motivations given by each of the patients. From this analysis, we determined that there was one cluster where individuals were mainly motivated to maintain physical activity for weight loss (Weight Loss group), one cluster which primarily focused on illness prevention such as diabetes and hypertension (Illness Prevention group), and one cluster that was mainly motivated by improving overall health (Health Promotion group). Overall, 19, 138, and 46 participants were classified to the Weight Loss group, the Illness Prevention and the Health Promotion groups. Table 2 shows the results comparing baseline sociodemographic characteristics and cardiovascular risks among these 3 groups. The Weight Loss group was significantly younger than the Illness Prevention and Health Promotion groups (overall *P*<.001). The racial and ethnic distribution also significantly differed among the 3 groups (*P*=.002). The Illness Prevention group has a larger number of Caucasians compared to the Weight Loss group (*P*=.001), while the Weight Loss group tended to be composed mostly of those who identified as African American, Hispanic, or mixed race compared to the Illness Prevention and Health Promotion groups (*P*=.008, *P*=.006, respectively). Additionally, the Health Promotion group tended to have lower BMI scores compared to the Illness Prevention group (overall *P*=.02). No other significant differences at the 95% confidence level were found across the remaining sociodemographic and cardiovascular risk factors (overall *P*>.05; see Table 2). The baseline moderate-to-vigorous intensity activity level did not differ among the 3 groups (overall *P*>.05).

**Table 1:** Use of digital technology and physical activity at 12 months after the intervention. The presence of two footnotes indicate a pairwise comparison.

Digital technology and activity		Overall (N=203), n (%)		Control (n=69), n (%)		Regular (n=69), n (%)		Plus (n=65), n (%)		Overall *P* value	
Do you currently have a health-related mobile app? (Yes)		84 (41.4)		29 (42.6)		30 (44.1)		25 (39.1)		.94	
Do you currently wear a pedometer? (Yes)		84 (41.4)		18 (26.1)^a,b^		36 (52.2)^a^		30 (46.2)^b^		.01	
Do you have your own pedometer? (Yes)		125 (61.9)		35 (51.1)		47 (68.1)		43 (66.2)		.09	
**Types**										.50	
	Fitbit		50 (24.6)		11 (5.4)		23 (11.2)		16 (7.9)		
	Omron		26 (12.8)		7 (3.5)		8 (3.9)		11 (11.2)		
	Other		23 (11.2)		11 (5.4)		8 (3.9)		7 (3.5)		
	Do not know		26 (12.8)		6 (3.0)		8 (3.9)		9 (4.4)		
Do you have your own pedometer? (No)		78 (38.1)		34 (48.9)		22 (31.9)		22 (33.8)			
**Reasons for not purchasing**											.17
	Still planning to purchase/keep looking		28 (13.8)		13 (6.4)		9 (4.4)		6 (3.0)		
	Too expensive/financial difficulty		17 (8.4)		2 (1.0)		9 (4.4)		6 (3.0)		
	Use app/phone/be able to estimate steps		9 (4.4)		4 (2.0)		1 (0.5)		4 (2.0)		
	Do not help/do not like		7 (3.5)		5 (2.5)		0 (0)		2 (1.0)		
	Technology challenging/not accurate		6 (3.0)		4 (2.0)		1 (0.5)		1 (0.5)		
	Has one somewhere/hasn’t set up		3 (1.5)		2 (1.0)		0 (0)		1 (0.5)		
	Other		5 (2.5)		2 (1.0)		1 (0.5)		2 (1.0)		
**Since your 9-month visit, what types of exercise have you engaged in to be physically active? (multiple choice question)**											
	Walking		126 (62.1)		49 (71.0)		44 (63.8)		33 (50.8)		.05
	Brisk walking		94 (46.3)		21 (30.4)^a,a^		35 (50.7)^a^		38 (58.5)^a^		.003
	Yoga		20 (9.9)		3 (4.3)		7 (10.1)		10 (15.4)		.10
	Hiking		15 (7.4)		5 (7.2)		4 (5.8)		6 (9.2)		.75
	Gardening/Yard work		16 (7.9)		7 (10.1)		3 (4.3)		6 (9.2)		.40
	Cycling		19 (9.4)		7 (10.1)		5 (7.2)		7 (10.8)		.75
	Other		110 (54.2)		39 (56.5)		35 (50.7)		36 (55.4)		.77
**Since your 9-month study visit, has your physical activity been more, less, or about the same (compared to the first 9 months of the study)?**										.006	
	More		64 (31.5)		29 (42.0)^c^		16 (23.2)^c^		19 (29.2)		
	Less		73 (36.0)		13 (18.8)^a,d^		33 (47.8)^a^		27 (41.6)^d^		
	About the same		66 (32.5)		27 (39.2)		20 (29.0)		19 (29.2)		
**Top 3 reasons for being less active after the 9-month visit (multiple choice question)**		**n=73**		**n=13**		**n=33**		**n=37**			
	Study ended		20 (27.4)		0 (0)		12 (16.4)		8 (11.0)		.04
	Lack of time		20 (27.4)		4 (5.8)		9 (13.0)		7 (10.8)		.02
	Did not have a pedometer		12 (16.4)		2 (2.7)		3 (4.1)		7 (9.6)		.21

^a^*P*<.001.

^b^*P*=.008.

^c^*P*=.009.

^d^*P*=.002.

**Table 2:** Baseline characteristics of participants by 3 cluster groups. The presence of two footnotes indicate a pairwise comparison.

Demographic^a^					Weight Loss group (n=19)		Illness Prevention group (n=138)		Health Promotion group (n=46)		Overall *P* value
Age (years), mean (SD)					41.5 (12.0)^b,b^		53.9 (10.4)^b^		53.2 (9.7)^b^		<.001
**Race/Ethnicity, n (%)**											
	White				4 (21.1)^c^		87 (63.0)^c^		24 (52.2)		.002
	Asian				5 (26.3)		22 (15.9)		14 (30.4)		
	African American, Hispanic, mixed				10 (52.6)^d,e^		29 (21.0)^d^		8 (17.4)^e^		
**Education, n (%)**											
	Completed high school and some college				6 (31.6)		34 (24.6)		11 (23.9)		.43
	Completed college				6 (31.6)		62 (44.9)		15 (32.6)		
	Completed graduate school				7 (36.8)		42 (30.4)		20 (43.5)		
**Marital Status, n (%)**											
	Currently married/cohabitating				8 (42.1)		75 (54.3)		23 (50.0)		.57
**Employment, n (%)**											
	Employed for pay (full or part time)				14 (73.7)		100 (72.5)		37 (80.4)		.56
**Cardiovascular risk factors**											
		Body mass index (kg/m^2^), mean (SD)		31.2 (6.9)		30.4 (6.0)^f^		27.7 (5.8)^f^		.02	
	**Smoking Status, n (%)**										
			Current smoker		1 (5.3)		2 (1.4)		1 (2.2)		.53
Menopause, n (%)					6 (31.6)^g^		88 (63.8)^g^		27 (58.7)		.03
High blood pressure, n (%)					3 (15.8)		50 (36.2)		15 (36.2)		.21
High total cholesterol, n (%)					4 (21.1)		51 (37.0)		14 (30.4)		.33
High glucose Diabetes, n (%)					3 (15.8)		10 (7.2)		3 (6.5)		.40
CESD score>16 points or taking antidepressant, n (%)					5 (26.3)		48 (34.8)		14 (30.4)		.70

^a^For the continuous variables, the mean and standard deviation, minimum, and maximum are shown; P value is based on ANOVA test. For categorical variables, frequency and percent are shown, where percentages are computed based on the number of non-missing observations in each treatment group and overall; P value is based on Chi-square test or Fisher exact test. Pairwise between-group differences with *P*<.05 and Bonferroni adjustment were used to control for multiple comparisons

^b^*P*<.001

^c^*P*=.001

^d^*P*=.007

^e^*P*=.01

^f^*P=*.03

^g^*P*=.027

## Discussion

### Principal Results

The present study aims to describe utilization of digital technologies and physical activity engagement post intervention, and to identify motivational profiles using NLP and clustering techniques in women who completed the mPED trial. We demonstrated the value of the use of NLP for participants’ responses to an open-ended question. NLP and cluster analysis resulted in 3 distinguished clustering groups that were labeled as 1) the Weight Loss group, 2) the Illness Prevention group, and 3) the Health Promotion group. [16-20] In a recent study of applying NLP to EHR to automatically assess delivery of weight management counseling in two regions of Kaiser Permanente, it was demonstrated that NLP had similar capabilities as trained medical record abstractors [16]. Additionally, use of a Wikipedia data dump in our NLP analysis in this paper was supported by the study finding by Ramesh and colleagues in 2013 that Wikipedia, compared to MedlinePlus and the Unified Medical Language System, significantly improved EHR note readability [19]. Thus, NLP appears to offer an effective way to classify short free texting interview data.

Several studies examined physical activity motivational profiles using cluster analysis techniques [32-41], but the clear majority of these studies targeted children and college students and used the Self-Determination Theory. In addition, none of these studies applied NLP in their studies. Therefore, it is difficult to make head-to-head comparisons with those studies in terms of characteristics of the cluster groups. While our study applied NLP to female participants’ responses to an open-ended question, the previous studies used a questionnaire in a sample of both men and boys and women and girls [32-34,36-40]. For example, in the cluster analysis study of profiling physical activity motivation based on the Exercise Self-Regulation Questionnaire in a large adult sample participating in a physical activity study, 3 cluster groups (the low motivation, controlled motivation, and autonomous motivation groups) were identified. The autonomous motivation group, representing 53% of the sample, had a higher level of education and a lower BMI than the other 2 groups [32]. Race and ethnicity for the groups was not reported in the study.

It is important to note that in this study, 3 cluster groups were identified, but overall the characteristics of the Weight Loss group differed considerably from the other 2 groups, and the Weight Loss group represent only a small proportion of the sample (19/203). A much higher number of younger women and African American, Latino, or mixed-race women were in the Weight Loss group. These study findings are like our previous focus group study findings that physical appearance was not a big motivator for healthy eating in most participants, especially the older ones [42]. The most frequently reported motivation was to imagine unwanted outcomes from bad eating habits, such as a heart attack and diabetes [42]. We believe that understanding an individual’s motivation is important because it helps clinicians and researchers tailor a physical activity maintenance intervention for women. Additionally, previous systematic reviews suggest one’s motivation plays a critical role in sustaining physical activity after the intervention, and tailoring the intervention significantly improves adherence [43,44].

Lastly, it is encouraging that even after all subjects returned the study accelerometer and stopped accessing the study app (if any) at 9 months, approximately half of the 2 intervention groups (Regular and Plus) reported still wearing an accelerometer and engaging in brisk walking as they were directed during the intervention phases. These numbers in the 2 intervention groups were much higher than the Control group. In contrast, a much higher proportion of the sample in the 2 intervention groups reported that they became less active than the Control group since the last research office visit. This finding is probably due to the small increase of physical activity in the Control group during the 9-month study period, while a substantial increase in physical activity was observed in the intervention groups [21-23]. We could assume that the intervention groups were less active in the 3 months post-study than they were during the first 9 months of the study period itself, but their level of physical activity engagement was probably still greater than the Control group. However, as we demonstrated in our previous report [23], without objectively measured physical activity data, this assumption could not be confirmed.

### Strengths and Limitations

Although to the best of our knowledge, this was the first study to examine physical activity maintenance motivational profiles using NLP and cluster analysis, several limitations need to be acknowledged. First, the sample represents only physically inactive female adults. The findings may not be generalizable to men or children, and physical activity engagement post intervention might be overestimated due to self-reported measures. Second, because this study was an exploratory investigation limited to the 12-month cross-sectional data, any causal relationship cannot be established. Third, the bag-of-words model that was used in this study for NLP tasks does not take into consideration the order in which words appear in a sentence, nor does it take into consideration part of speech labels. The strength of the bag-of-words model is that it can generate insights based on frequently occurring combinations of words. In addition, we note that word-vectors produced by the word2vec model cannot be easily interpreted, and that the effectiveness of these vectors for classification and clustering is dependent on hyper-parameters such as the word-vector dimension. However, the word2vec model has the advantage that it preserves semantic and synthetic relationships from the original text [45]. Similarly, the K-means cluster analysis used in this study is an unsupervised method which identifies patterns using criteria only based on data and not ground truth labels, and it is sensitive to the total number of clusters used. We used the elbow criterion [29] to mitigate the sensitivity in our analysis to the number of clusters.

### Conclusion

The motivation profiles for being physically active post-intervention was classified into three cluster groups: The Weight Loss group; the Illness Prevention group; and the Health Promotion group. The Weight Loss Group differed considerably from the other two groups. This information could be relevant to tailoring a physical activity maintenance intervention. Furthermore, the findings from NLP and cluster analysis are useful methods to analyze short free text to differentiate motivational profiles. As more sophisticated NLP tools are developed in the future, the potential of NLP applications in behavioral research will broaden.

## Abbreviations

RCT: randomized controlled trial
BMI: body mass index
NLP: natural language processing
PCA: principal component analysis
mPED: mobile phone—based physical activity education
